# First report of a comparative patient-oriented perspective on the use of non-vitamin-K oral anticoagulants or vitamin-K antagonists in atrial fibrillation: patients’ experiences, side-effects and practical problems leading to non-adherence

**DOI:** 10.1007/s12471-019-01331-x

**Published:** 2019-11-19

**Authors:** N. Bennaghmouch, A. J. W. M. de Veer, C. Zivelonghi, L. van Dijk, J. M. ten Berg

**Affiliations:** 1 Department of Cardiology, St. Antonius Hospital, Nieuwegein, The Netherlands; 2 Department of Pharmaceutical Care, Netherlands Institute for Health Services Research (NIVEL), Utrecht, The Netherlands

**Keywords:** Anticoagulation, Atrial fibrillation, Adherence

## Abstract

**Background:**

Non-vitamin‑K oral anticoagulants (NOACs) are recommended as the first-choice therapy for stroke prevention in patients with non-valvular atrial fibrillation (AF). However, the lack of monitoring may impact patients’ adherence, and non-adherence to medication is a potential hazard to safe and efficacious use. This is the first report with a ‘comparative patient-oriented perspective’ regarding the use of anticoagulant medication in the NOACs era. Our aim was to compare patients’ self-reported practical problems, adverse events and non-adherence to anticoagulation therapy.

**Methods:**

A survey was conducted among patients with AF on either NOACs or vitamin‑K antagonists (VKAs). The outcomes were self-reported non-adherence to anticoagulant medication, and patients’ experiences, adverse events and practical problems correlated with the intake of the drug itself.

**Results:**

A total of 765 patients filled out the questionnaire, of which 389 (50.9%) were on VKAs and 376 (49.1%) on NOACs. Age (70.6 ± 8.8 vs 70.3 ± 9.1 years) and male gender (70.4% vs 64.6%) were similar in the two groups. A significantly higher proportion of VKA users than NOAC users reported having frequent (16.2% vs 3.7%, *p* > 0.001) or occasional (4.1% vs 1.3%, *p* > 0.001) practical issues with medication intake. Self-reported non-adherence was significantly higher (24.4% vs 18.1%, *p* = 0.03) among VKA users. The incidence of self-reported adverse events was similar.

**Conclusion:**

Patient experiences support the current guideline recommendations for NOACs as the first-choice therapy: NOAC therapy resulted in a higher practical feasibility and better adherence when compared with VKA therapy, with a similar incidence of adverse events in both groups.

**Electronic supplementary material:**

The online version of this article (10.1007/s12471-019-01331-x) contains supplementary material, which is available to authorized users.

## What’s new?

- We report and compare practical problems, adverse events and non-adherence to anticoagulation therapy from a patient-oriented perspective.
- A significantly higher proportion of VKA as compared with NOAC users reported having practical issues with the intake of the medication and reported being non-adherent.
- This study supports the current guideline recommendations for NOACs as the first-choice therapy for stroke prevention in atrial fibrillation patients.

## Introduction

Atrial fibrillation (AF) is associated with increased mortality and morbidity [1]. Previously, AF patients with at least one risk factor for stroke (e.g. age >65 years, congestive heart failure, hypertension, diabetes, prior stroke/transient ischaemic attack, vascular disease) were usually treated with a vitamin‑K antagonist (VKA) for stroke prevention. These anticoagulant medicines require intensive monitoring as their effect can fluctuate. With the recently introduced non-vitamin‑K oral anticoagulants (NOACs), a new and more practical alternative to VKAs has been introduced. Several advantages of NOACs, such as no need for INR monitoring, fixed daily doses and only a few interactions with food and medication, have resulted in increased use in daily practice. Recent guidelines for the management of patients with AF have endorsed NOACs as a class IA recommendation [2–4]. Features of NOACs are a better safety and an at least similar efficacy profile when compared with VKAs [5–8]. On the other hand, the lack of monitoring may predispose patients to non-adherence, and non-adherence to medication is a potential hazard to the safe and efficacious use of NOACs. Medication adherence is defined as the accurate intake of medications based on the dose, frequency and schedule prescribed [9]. Although NOACs are being increasingly prescribed, there are still many patients who use VKAs, especially in the Netherlands, our study setting. The Netherlands has a national infrastructure of anticoagulant services in place to monitor VKA users.

Limited data exist on AF patients’ experiences and perceptions of taking NOACs in comparison to VKAs for stroke prevention. The purpose of this study is to evaluate patients’ experiences, practical problems, adverse events and non-adherence to anticoagulation therapy with NOACs and VKAs. This is the first study on patient self-reported experiences with anticoagulation therapy in the NOAC era.

## Methods

This is a multi-centre prospective study assessing the perspective and self-reported adherence of AF patients to an anticoagulation regimen for stroke prevention. For this purpose, a specifically designed questionnaire developed by NIVEL, the Netherlands Institute for Health Services Research, was used, based on questionnaires previously used for other medicines [10, 11]. Self-reported adherence was measured using the Dutch version of the validated Morisky medication adherence scale (MMAS-8) [12]. This questionnaire was made specifically for anticoagulants.

Patients with AF on either VKAs or NOACs were invited to participate. Among them were both experienced and new anticoagulation drug users. No ICD or DBC (*Diagnose Behandel Combinatie*; English: diagnosis treatment combination) codes were used for screening. The patients using VKAs invited to participate were recruited through the Star-MDC (Star Medical Diagnostic Centre) Rotterdam and the patients using NOACs through the St Antonius Hospital Nieuwegein for the VKA group and NOAC group, respectively. The St. Antonius Hospital approached the first 1200 NOAC users in their hospital. The Star-MDC is an anticoagulation clinic where patients treated with VKAs are monitored. The St Antonius Hospital houses a tertiary cardiology clinic which is specialised in antithrombotic therapy. Patients were requested, online or on paper, to fill in their own individual (patient) data. The study was conducted from August 2016 to December 2016.

The study received institutional review board approval from the Medical Ethics Review Committee of VU University Medical Centre Amsterdam (protocol 2016.108). All patients provided informed consent prior to filling out the questionnaire.

### Endpoints

The primary endpoint of this analysis comprised eight items for self-reported non-adherence to anticoagulant medication due to any medication-related issue measured with the MMAS‑8. Additional outcomes were self-reported adverse events and practical problems relating to the intake of the medication.

In addition, participants were further questioned on the incidence of several categories of non-adherence: occasional lack of adherence, lack of compliance in therapy, lack of adherence because of practical problems, not having taken the anticoagulant the previous day and lack of adherence due to an absence of symptoms. The practical problems studied consisted of problems with the size and shape of the drug, intake issues, and problems with dose adjustment according to the intake schedule. Lastly, we studied the patient-reported incidence of allergic reactions, haematomas and bleeding as well as the subsequent level of severity.

### Statistical analysis

Descriptive analysis of the data was performed using summary statistics for categorical and quantitative (continuous) data. Continuous data were described as medians with interquartile range or means with standard deviation. Categorical data were expressed as percentages. Distributions of categorical data were examined by the *X*^2^-test or Fisher’s exact test, as appropriate. Continuous data were compared using Student’s *t*-test or the Mann-Whitney U test, as appropriate. The analyses were performed using SPSS software for Windows, version 22 (IBM Corporation, Armonk, NY, USA).

## Results

### Patient characteristics

**Fig. 1:**
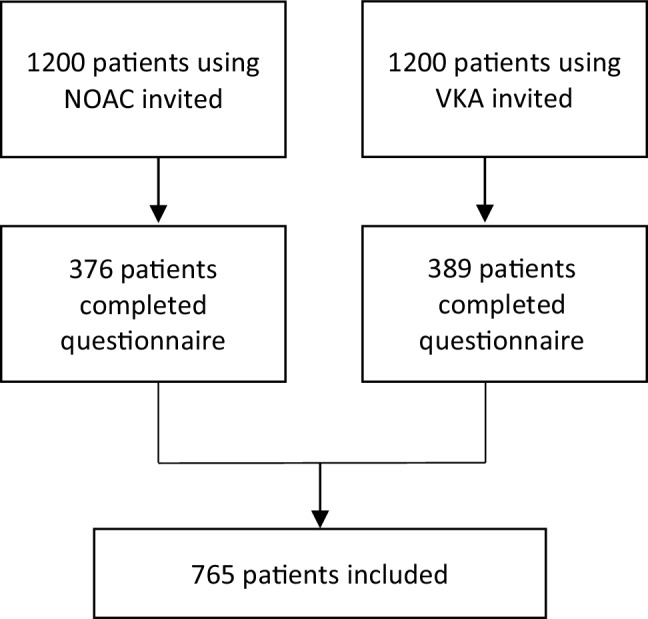
Flowchart of the study population. *NOAC* non-vitamin‑K oral anticoagulants, *VKA* vitamin‑K antagonists

In total, 2400 patients were invited to fill in the questionnaire. A response rate of ±32% was achieved, resulting in 765 completed questionnaires. A flowchart of the study population is presented in Fig. 1. Of the patients studied, 389 (50.9%) were on a VKA and 376 (49.1%) on a NOAC. At baseline, mean age was similar in the two groups (70.6 ± 8.8 vs 70.3 ± 9.1 years), as was male gender (70.4% vs 64.6%, *p* = 0.13). The self-reported history of coronary artery disease and stroke/transient ischaemic attack was similar in the two groups (10.8% vs 9%, *p* = 0.47, and 7.5% vs 6.9%, *p* = 0.78, respectively). A self-reported history of thrombosis and a history of embolism were reported more frequently in the VKA group (8.7% vs 3.2%, *p* = 0.001, and 4.9% vs 1.3%, *p* = 0.006, respectively). A significantly higher prevalence of use of antidiabetic medication (16.2% vs 9.8%, *p* = 0.01) was reported in the VKA group. Furthermore, a lower prevalence of use of P2Y12 inhibitors (1.3% vs 5.6%, *p* = 0.001) was reported in the VKA group. Also, polypharmacy (>3 drugs) was reported more frequently in the VKA group (53.5% vs 45.7%, *p* = 0.04). All baseline variables are shown in Tab. 1.

**Table 1:** Patient demographics

		VKA (*n* = 389)	NOAC (*n* = 376)	*p*-value
Age, mean ± SD		70.6 ± 8.8	70.3 ± 9.1	0.68
Male, *n *(%)		209 (70.4%)	235 (64.6%)	0.13
Type of NOAC				NA
	Apixaban, *n *(%)	NA	58 (15.4%)	
	Dabigatran, *n *(%)	NA	24 (6.4%)	
	Rivaroxaban, *n *(%)	NA	291 (77.4%)	
	Edoxaban, *n *(%)	NA	3 (0.8%)	
Previous thrombosis, *n *(%)		34 (8.7%)	12 (3.2%)	0.001
Previous embolism, *n *(%)		19 (4.9%)	5 (1.3%)	0.006
CAD, *n *(%)		42 (10.8%)	34 (9%)	0.47
Previous stroke/TIA, *n *(%)		29 (7.5%)	26 (6.9%)	0.78
Additional medical therapy				
	ASA, *n *(%)	10 (2.6%)	3 (0.8%)	0.09
	P2Y12 inhibitor, *n *(%)	5 (1.3%)	21 (5.6%)	0.001
	Antidiabetic drugs, *n *(%)	63 (16.2%)	37 (9.8%)	0.01
	Antidepressants, *n *(%)	10 (2.6%)	15 (4%)	0.31
	Polypharmacy (>3), *n *(%)	208 (53.5%)	172 (45.7%)	0.04

*VKA* vitamin‑K antagonist, *NOAC* non-vitamin‑K oral anticoagulant, *CAD* coronary artery disease, *TIA* transient ischaemic attack, *ASA* acetylsalicylic acid

### Patient adherence

The primary endpoint of our analysis, namely any sort of lack of adherence to anticoagulation medication, occurred in 95 (24.4%) patients in the VKA group versus 68 (18.1%) in the NOAC group (*p* = 0.03). Detailed categories of non-adherence are reported in Tab. 2. More VKA users than NOAC users reported forgetting their medication in the previous 2 weeks (question 2); however, this difference was not statistically significant. Also, more VKA users than NOAC users reported having taken their medication the day before (question 3). Furthermore, more patients in the VKA group reported finding the use of anticoagulation more ‘cumbersome’ (8% vs 4.5%, respectively, *p* = 0.05).

**Table 2:** Self-reported non-adherence in VKA users and NOAC users

		VKA (*n* = 389)	NOAC (*n* = 376)		*p*-value
*Any category of non-adherence*		95 (24.4%)	68 (18.1%)		0.03
1. Do you sometimes forget to take your medication?		60 (15.4%)	37 (9.8%)		0.02
2. People sometimes miss a dose of their medication for reasons other than forgetting. Over the past 2 weeks, were there any days on which you did not take your medication?		5 (1.3%)	12 (3.2%)		0.09
3. Have you ever cut back or stopped taking your medication without telling your doctor because you felt worse when you took it?		9 (2.3%)	7 (1.9%)		0.80
4. When you travel or are away from home, do you sometimes forget to take your medication?		13 (3.3%)	12 (3.2%)		1
5. Did you take all your medication yesterday?		34 (8.7%)	25 (6.6%)		0.34
6. When you feel like your symptoms are under control, do you sometimes stop taking your medication?		3 (0.8%)	8 (2.1%)		0.14
7. Taking medication every day is a real inconvenience for some people. Do you ever feel hassled about sticking to your treatment plan?		31 (8%)	17 (4.5%)		0.05
8. How often do you have difficulty remembering to take your medication?	Never	341 (87.7%)	349 (92.8%)		0.41
	Once in a while	33 (8.5%)	20 (5.3%)		
	Sometimes	14 (3.6%)	4 (1.1%)		
	Usually	1 (0.3%)	2 (0.5%)		
	All the time	0 (0%)	1 (0.3%)		

*VKA* vitamin‑K antagonist, *NOAC* non-vitamin‑K oral anticoagulant

### Practical issues

Practical issues regarding medication intake were reported more frequently in the VKA group than in the NOAC group. Practical issues included having problems with pill size and shape, having to split the pill, and being required to follow a variable intake schedule when using a VKA. Details regarding patient-reported practical issues in the two treatment groups are presented in Tab. 3.

**Table 3:** Self-reported incidence of practical issues in VKA and NOAC users

		VKA (*n* = 389)	NOAC (*n* = 376)	*p*-value
*Patients reporting practical issues*				<0.001
	Common	63 (16.2%)	14 (3.7%)	
	Occasional	16 (4.1%)	5 (1.3%)	
*Size and shape of drug*				0.001
	Common	31 (8%)	9 (2.4%)	
	Occasional	5 (1.3%)	1 (0.3%)	
Level of hindrance				
	Moderate	9(26.5%)	2 (22.2%)	
	Serious	12 (35.3%)	2 (22.2%)	
	Severe	12 (35.3%)	5 (55.6%)	
*Intake issues*				0.14
	Common	10 (2.6%)	3 (0.8%)	
	Occasional	2 (0.5%)	1 (0.3%)	
Level of hindrance				
	Moderate	5 (45.5%)	2 (66.7%)	
	Serious	3 (27.3%)	1 (33.3%)	
	Severe	3 (27.3%)	0	
*Dose adjustment according to scheme*				<0.001
	Common	32 (8.2%)	4 (1.1%)	
	Occasional	10 (2.6%)	3 (0.8%)	
Level of hindrance				
	Moderate	19 (45.2%)	3 (42.9%)	
	Serious	7 (16.7%)	1 (14.3%)	
	Severe	7 (16.7%)	2 (28.6%)	

Complete questions (in Dutch) available in the Electronic Supplementary Material, Appendix 1

*VKA* vitamin‑K antagonist, *NOAC* non-vitamin‑K oral anticoagulant

### Adverse events

The self-reported incidence of adverse events was similar in the two groups. These results are shown in Table S1 (Electronic Supplementary Material).

## Discussion

To the best of our knowledge, our study is the first to compare self-reported adherence to NOAC and VKA therapy in a ‘real-world’ population with AF and an indication for oral anticoagulation therapy. The present study yielded three main findings: (1) adherence to medication is significantly higher in patients using NOACs than in those using VKAs; (2) the rate of practical issues experienced was significantly higher among VKA users than NOAC users; and (3) the self-reported incidence of adverse events was similar in the two groups.

### Non-adherence

Non-adherence to medications has been documented to occur in >60% of cardiovascular patients [13]. With the introduction of the NOACs in the Netherlands, questions were raised concerning the non-adherence to regimens for these new drugs in daily practice. The argument was that by eliminating the frequent visits to the anticoagulation clinic, the rate of adherence would decrease [14]. The use of drugs in a clinical trial setting differs from that in real-world populations, as randomised controlled trials (RCTs) generally have stricter monitoring schemes and more frequent follow-up visits, which enhances medication adherence. A previous database study evaluated NOAC treatment persistence rates, in which non-persistence was defined as a >14-day gap between prescriptions [15]. The follow-up period was 6 months and approximately one-third of patients were non-persistent with dabigatran or rivaroxaban therapy. However, our analysis shows that self-reported non-adherence is significantly lower in NOAC users than in VKA users. Sørensen et al. also showed lower non-adherence in NOAC users; however, the non-adherence was not measured by self-reporting but by linking Danish nationwide registries to prescription registries and then calculating the proportions of days covered [16]. Improved adherence to NOAC therapy may be the result of a lower monitoring burden and fewer food and drug interactions as compared with VKAs. Conversely, it also could be pointed out that adherence to NOAC therapy could be due less to the lack of visits to the coagulation clinic, with fewer chances to highlight the importance of treatment adherence. Good adherence to NOAC therapy is an important aspect with regard to their safety and effectiveness. Consequently, improving medication adherence could lead to a cost reduction in the public health sector by reducing the number of events [17]. Since anticoagulation drugs have a preventive mechanism that does not make symptom relief noticeable to patients, they might be less likely to take the medication. So, procedures to improve adherence are imperative. According to the European guidelines, the European Heart Rhythm Association NOAC card should be distributed to patients on NOACs at the initiation of therapy and during follow-up [2]. Furthermore, to ensure good adherence to the prescribed OAC regimen patient-tailored approaches (e.g. pill box, calendar, electronic reminders) should be used.

### Practical issues

Self-reported practical problems with the use of NOACs and VKAs have not been studied previously. We report a significant difference in favour of NOAC therapy. As expected, the patients reporting problems with following their dosing scheme were more frequently VKA users. Given the narrow therapeutic window (INR-adjusted range: 2–3) of VKAs, dose adjustment is often necessary. Therefore, some VKA users need to take a different number of pills every day. Having practical issues with the size and shape of the drug is also reported more frequently by VKA users. These included having difficulty opening the blister pack, changes in the packaging and name of the drug, and the drug looking similar to other drugs they are taking. NOAC users might be experiencing these problems less frequently, since these drugs are still under patent, so the packaging and name will not change. Examples of problems reported concerning the intake of VKAs are tablets accidentally crumbling when dispensing and tablets being too small. We have not investigated to what extent the reported practical problems have led to non-adherence.

### Adverse events

This survey provides some insights into the self-reported incidence of adverse events in a real-world population of AF patients using NOACs. Participants also reported the level of severity of these adverse events. As compared with the pivotal RCTs comparing NOACs with VKAs, there does not seem to be a large difference in the reported incidence of bleeding, haematomas and allergic reactions. The Apixaban for Reduction in Stroke and Other Thromboembolic Events in Atrial Fibrillation (ARISTOTLE) study reported an annual incidence of any bleeding of 18.1% and 25.8% for apixaban and warfarin, respectively [hazard ratio (HR) 0.71, confidence interval (CI) 0.68–0.75, *p* < 0.001] [7]. Furthermore, the Effective Anticoagulation with Factor Xa Next Generation in Atrial Fibrillation—Thrombolysis in Myocardial Infarction 48 (ENGAGE AF-TIMI 48) trial reported an annual incidence of any overt bleeding of 14.15% and 16.40% for high-dose edoxaban and warfarin, respectively (HR 0.87, CI 0.82–0.92, *p* < 0.001) [8]. The Rivaroxaban Once Daily Oral Direct Factor Xa Inhibition Compared with Vitamin K Antagonism for Prevention of Stroke and Embolism Trial in Atrial Fibrillation (ROCKET-AF) and Randomised Evaluation of Long-Term Anticoagulant Therapy (RE-LY) trials did not report on the incidence of any bleeding [5, 6]. However, since it concerns self-reported events, a comparison with adverse events reported in RCTs and registries is not completely feasible. But even more importantly, the self-reported incidence of these adverse events is similar in both NOAC and VKA users. It is therefore unlikely that the difference in self-reported non-adherence can be explained by experiencing adverse effects from NOAC or VKA use.

### Strengths and limitations

Our study has various strengths. First, the development of the questionnaire was based on a thorough review of the literature and findings from focus groups. Second, with regard to adverse events, we addressed not only rates of adverse effects, but also the self-reported level of severity. Third, there was ample variation in the patients’ demographic characteristics.

Our study is also subject to limitations. First, because the questionnaire was anonymous, only self-reported baseline characteristics could be collected. Moreover, we could not collect follow-up data of the participants. And the understanding of various terms, such as thrombosis and embolism, might have been interpreted differently by patients. Second, we do not have any information available regarding the anticoagulant users who did not fill out the questionnaire. Thus, it is possible that patients not responding to the questionnaire have a worse medication adherence than those who participated in our study. On the other hand, due to the self-reporting character, the data provided by the patients may have been more favourable than in reality. However, it is plausible that these proportions are similar in NOAC and VKA users. Third, the settings for data collection differed for VKAs and NOACs. While VKA patients were recruited through an anticoagulant clinic, the NOAC users were approached by a specialised hospital. Moreover, only one centre recruited patients for the VKA and NOAC group, respectively. The care provided in these centres may have an impact on patient experiences. For example, if the care in these centres is more patient-oriented than in other centres, adherence levels may be higher than in the overall population. Therefore, it would be worthwhile to use the questionnaire in other centres. Fourth, the populations who use VKAs and NOACs are different, so a complete comparison is not possible. For instance, polypharmacy was more frequent in the VKA than in the NOAC group. A relationship might exist between the number of drugs used and adherence. Furthermore, we did not collect data on renal dysfunction. Renal dysfunction might be a confounding factor since prescribers might refrain from prescribing NOACs to patients with renal dysfunction. Fifth, measuring non-adherence is a challenge. Previously used methods are obtaining medication adherence data by linking refill gaps and prescription data to existing registries and electronic monitoring. As patient experience influences medication intake and consequently medication adherence, we believe centralising the patient by using self-reported data on their experiences and adherence is appropriate. Still, it would be interesting to study patients’ experiences related to more objective measurements of adherence. Finally, the MMAS‑8 questionnaire has some shortcomings. It is not clear whether ‘sometimes’ forgetting medication is once a year or once a week, for example. Furthermore, some MMAS‑8 questions do not provide hard evidence for non-adherence.

In conclusion, in this first real-world report of a ‘fully patient-oriented perspective’ on the use of anticoagulant medication in the NOAC era we demonstrated that the use of NOACs resulted in a higher practical feasibility and consequent adherence profile when compared with VKAs. Furthermore, the reported incidence of side-effects was similar in both groups. These findings support the guideline recommendation for NOACs as the first-choice anticoagulant therapy for stroke prevention in non-valvular AF patients.

## Caption Electronic Supplementary Material

Appendix 1 – Questionnaire | questions on practical issues (in Dutch); Appendix 2 – Table S1 | Adverse events reported in VKA-users and NOAC-users
