# Correction to: First report of a comparative patient-oriented perspective on the use of non-vitamin-K oral anticoagulants or vitamin-K antagonists in atrial fibrillation: patients’ experiences, side-effects and practical problems leading to non-adherence

**DOI:** 10.1007/s12471-021-01577-4

**Published:** 2021-04-16

**Authors:** N. Bennaghmouch, A. J. W. M. de Veer, C. Zivelonghi, L. van Dijk, J. M. ten Berg

**Affiliations:** 1 Department of Cardiology, St. Antonius Hospital, Nieuwegein, The Netherlands; 2 Department of Pharmaceutical Care, Netherlands Institute for Health Services Research (NIVEL), Utrecht, The Netherlands

**Correction to:**

**Neth Heart J 2019**

10.1007/s12471-019-01331-x

The original version of this article unfortunately contained a mistake. The authors of “First report of a comparative patient-oriented perspective on the use of non-vitamin‑K oral anticoagulants or vitamin‑K antagonists in atrial fibrillation: patients’ experiences, side-effects and practical problems leading to non-adherence brillation: patients’ experiences, side-effects and practical problems leading to non-adherence” (Neth Heart J (2019) 27:596–604) advise that in order to address concerns raised by the purported holders of intellectual property interests in the Morisky Medication Adherence Scale (“MMAS”), Appendices 1–3 that include the Morisky Medication Adherence Scale‑8 Item questionnaire should be removed from the article. As a result, these appendices were removed and the remaining appendices were renumbered. The original article has been corrected.

## Supplementary Information

Appendix 1 – Questionnaire | questions on practical issues (in Dutch); Appendix 2 – Table S1 | Adverse events reported in VKA-users and NOAC-users

