# Supplementary material for: First report of a comparative patient-oriented perspective on the use of non-vitamin-K oral anticoagulants or vitamin-K antagonists in atrial fibrillation: patients’ experiences, side-effects and practical problems leading to non-adherence
Source: Neth Heart J. 2019 Nov 19;27(12):596–604. doi: 10.1007/s12471-019-01331-x (PMC6890911; doi:10.1007/s12471-019-01331-x)
Supplement: Supplementary file 1 — Appendix 1 – Questionnaire | questions on practical issues (in Dutch); Appendix 2 – Table S1 | Adverse events reported in VKA-users and NOAC-users [file 12471_2019_1331_MOESM1_ESM.docx]

**Supplementary material**

*Bennaghmouch et al. First report on a comparative patient-oriented perspective about the use of NOAC or VKA in atrial fibrillation: patients’ experiences, side effects and practical problems leading to nonadherence.*

Appendix 1 – Questionnaire | questions on practical issues (in Dutch)

Appendix 2 – Table S1 | Adverse events reported in VKA-users and NOAC-users

1 – Questionnaire | practical issues (in Dutch)

| Heeft u in het afgelopen jaar één of meerdere praktische problemen bij het gebruik van bloedverdunners ervaren… | Ja | Nee |
| --- | --- | --- |
| 1. …in het algemeen? |  |  |
| 1. …met betrekking tot de verpakking en/of het uiterlijk van de medicatie? (Bijvoorbeeld tabletten moeilijk uit strip te krijgen, onleesbaar of onbegrijpelijk etiket, tabletten zien er hetzelfde uit als andere medicijnen) |  |  |
| 1. …met betrekking tot het formaat en/of de inname van de tabletten? (Bijvoorbeeld tabletten lastig te breken, tabletten moeilijk door te slikken, te kleine tabletten, onduidelijk hoe tabletten ingenomen moeten worden) |  |  |
| 1. …met betrekking tot het innemen van bloedverdunners volgens schema? (Bijvoorbeeld tabletten vergeten in te nemen, lastig om tabletten in te nemen door werk of vakantie) |  |  |

2 - Table S1 | Adverse events reported in VKA-users and NOAC-users

|  | | VKA (n=389) | NOAC (n=376) | p-value |
| --- | --- | --- | --- | --- |
| **Total adverse events incidence** | | 164 (46.6%) | 151 (44.4%) | 0.59 |
|  | | | | |
| **Allergic reactions** | | 17 (4.4%) | 20 (5.3%) | 0.61 |
| Level of severity | |  |  |  |
|  | Moderate | 6 (35.3%) | 10 (52.6%) |  |
|  | Serious | 4 (23.5%) | 3 (15.8%) |  |
|  | Severe | 6 (35.3%) | 4 (21.1%) |  |
|  | | | | |
| **Haematoma** | | 134 (37.6%) | 127 (36.3%) | 0.75 |
| Level of severity | |  |  |  |
|  | Moderate | 39 (29.5%) | 37 (30.1%) |  |
|  | Serious | 15 (11.4%) | 13 (10.6%) |  |
|  | Severe | 12 (9.1%) | 11 (8.9%) |  |
|  | | | | |
| **Bleedings** | | 62 (17.6%) | 64 (18.6%) | 0.77 |
| Level of severity | |  |  |  |
|  | Moderate | 22 (36.1%) | 22 (36.1%) |  |
|  | Serious | 11 (18%) | 13 (21.3%) |  |
|  | Severe | 15 (24.6%) | 14 (23%) |  |
